# Use of Rotational Atherectomy-Assisted Balloon Angioplasty in the Treatment of Isolated Below-the-Knee Atherosclerotic Lesions in Patients with Chronic Limb-Threatening Ischemia

**DOI:** 10.3390/jcm13051346

**Published:** 2024-02-27

**Authors:** Apostolos G. Pitoulias, Gergana T. Taneva, Konstantinos Avranas, Nizar Abu Bakr, Georgios A. Pitoulias, Konstantinos P. Donas

**Affiliations:** 1 Rhein Main Vascular Center, Department of Vascular and Endovascular Surgery, Asklepios Clinics Langen, Paulinen Wiesbaden, Seligenstadt, 63225 Langen, Germany; n.abubakr@asklepios.com (N.A.B.); konstantinos.donas@googlemail.com (K.P.D.); 2 Research Collaborator at Rhein Main Vascular Center, Department of Vascular and Endovascular Surgery, Asklepios Clinics Langen, Paulinen Wiesbaden, Seligenstadt, 63225 Langen, Germanyavranaskon@gmail.com (K.A.); pitoulias@yahoo.com (G.A.P.)

**Keywords:** PAD, rotational atherectomy, BTK-lesions, CLI

## Abstract

The aim of the study is to evaluate the safety and effectiveness of rotational atherectomy-assisted balloon angioplasty (BTK-RA) for the treatment of isolated below the knee (BTK) atherosclerotic lesions and to compare the outcomes to plain old balloon angioplasty (POBA). Between January 2020 and September 2023, 96 consecutive patients with chronic limb threatening ischemia (CTLI) and isolated BTK-lesions underwent POBA (group A) or BTK-RA (group B). The primary outcome measures were: periprocedural technical success, primary patency, postoperative increase of the ankle branchial index (ABI), target lesion revascularization (TLR), limb salvage, minor amputation and death. Both techniques had similar technical success, operative time, intraprocedural complications and bailout stent implantations, independently of the operator’s experience. Group B had significantly higher primary patency rates (93.5% vs. 72.0%, respectively, *p* = 0.006), TLR (2.1% vs. 24%, *p* = 0.057), lower in-hospital stay (2.0–3.0 vs. 4.0–6.0 days, respectively, *p* < 0.001) and higher postoperative ABI (0.8–0.2 vs. 0.7–0.1, respectively, *p* = 0.008), compared to group A. Significant differences (POBA *n*: 20, 40%, BTK-RA *n* = 3, 6.5%) were found in minor amputation rates between the two groups (*p* < 0.001), while the respective limb salvage rates were similar in both groups (94.0% vs. 97.8%, *p* = 0.35). The use of BTK-RA for the treatment of BTK-lesions in patients with CTLI showed significant clinical advantages in comparison to POBA.

## 1. Introduction

Peripheral artery disease (PAD), defined as an atherosclerotic flow limiting disease of the lower extremities, can be associated with increased cardiovascular mortality [1]. PAD affects 12–20% of the American population above 60 years old and currently limits the quality of life of 200 million patients worldwide [2,3], while encumbering national health systems in an ever-aging population. Despite the remarkable progress of medical and interventional technologies for the treatment of PAD, the BASIL study—which compares the long-term results of bypass as first therapy versus balloon angioplasty as first therapy for patients with severe limb ischemia—showed 56% mortality and 7% major amputation rates at a 5 year follow-up [4]. Data are even worse in chronic limb-threatening ischemia (CLTI), where the amputation rate rises to 40% after 6 months from presentation [5] and to 50% during the COVID-19 pandemic [6]. 

In the last decades, the endovascular treatment with plain old balloon angioplasty (POBA) has been established as a first line-treatment for CLTI patients with short stenotic lesions and occlusions in the infra-popliteal segment [7]. Since then, different atherectomy devices have been developed, proving their applicability in the femoropopliteal segment and even the common femoral artery (CFA) [8,9]. However, no robust conclusion can be drawn due to the plethora of atherectomy types and the absence of control groups. A recent study from Taneva et al. showed promising mid-term results for the use of rotational atherectomy assisted angioplasty in the treatment of femoropopliteal lesions with a primary patency rate of 97% at 12 months and 87% at 24 months [10]. However, the available data on the utilization of atherectomy in the infra-popliteal segment, specifically within the below-the-knee vessels, is notably limited [8,11]. Kumarasamy et al. [11] showed that rotational atherectomy for the treatment of BTK-lesions is feasible but did not provide any direct comparison with other endovascular techniques. This paucity of literature underlines the need for further research and exploration in this particular domain of vascular intervention. Understanding the efficacy and safety of atherectomy in BTK vessels is crucial for advancing our knowledge and improving treatment strategies for patients with peripheral artery disease affecting these lower extremity arteries. While evidence is scarce, the frequent occurrence of heavy calcification in tibial arteries significantly hampers the effectiveness of POBA, resulting in issues like dissections and recoil stenosis [12,13]. This highlights the potential benefits of vessel preparation through the debulking of heavily calcified plaques using rotational atherectomy beforehand.

Motivated by this gap in knowledge, our study aimed to assess the efficacy of below-the-knee rotational atherectomy in treating CLTI, comparing its outcomes to those of POBA. This research seeks to contribute valuable insights into the potential benefits of BTK-RA as an alternative or complementary approach in the management of this challenging condition. 

## 2. Materials and Methods

This single-center study was conducted as a non-sponsored retrospective analysis of prospectively collected data for patients with CTLI treated by endovascular means. The study protocol was approved by the institutional research committee and complied with the principles of the Declaration of Helsinki. Authors have no conflicts of interest to declare, and this study received no external funding.

### 2.1. Selection Criteria

Data of all consecutive patients with CLTI and an indication for BTK endovascular revascularization between January 2020 and September 2023 were analyzed. From January 2020 to December 2021 rotational atherectomy was not available in our institute and all consecutive patients enrolled during that time received POBA treatment in the BTK segment (group A). From January 2022 to September 2023, rotational atherectomy by the Phoenix device (Philips, US) became our standard approach and all consecutive patients during that period were enrolled in the BTK-RA group (group B). Patients with complex multi-level lesions affecting both the supra-popliteal vessels as well as the BTK-vessels were excluded. Demographic data, risk factors of PAD, preoperative and postoperative medication, the presence of occlusive disease, clinical status using the Rutherford classification and Ankle-Branchial Index (ABI) at presentation, discharge from hospital and during follow-up were recorded. ABI is a non-invasive test that compares the blood pressure in the upper and lower limbs. It is used for the diagnosis and assessment of PAD. An ABI < 0.9 is a strong indicator of PAD and is associated with increased risk of cardiovascular events while an ABI > 1.4 represents arterial stiffness due to medial calcification [7].

### 2.2. Endpoints

The primary endpoint was the evaluation of periprocedural technical success and primary patency rate. The secondary endpoints included target lesion revascularization (TLR), secondary patency, postoperative increase of ABI, limb salvage, minor amputation and death.

### 2.3. Definitions

Technical success was defined as the presence of <30% residual stenosis without signs of flow limiting dissection, peripheral embolization and/or signs of vessel perforation at completion of the initial procedure. Assisted technical success was achieved by fulfillment of the aforementioned criteria with implantation of a bailout stent. In case of primary patency failure during follow-up, the decision for reintervention was clinically driven, considering the presence of rest pain and wound or the need of limb salvage intervention. The preservation of the ankle and heel was characterized as limb salvage. Complications at the access site encompassed the occurrence of clinical and hemodynamic hematomas and aneurysm spurium.

### 2.4. Procedure

All procedures were performed with a C-arm (Ziehm Vision RFD Hybrid Edition) in supine position. Ipsilateral antegrade percutaneous approach was performed with 5F Terumo or Cordis introducer sheath (length of 55 cm) in all cases. The procedures were performed by vascular surgeons, certified by the German Society of Vascular Surgery as endovascular specialists, as well as by vascular surgeons who were not certified as endovascular specialists. After systemic heparinization with 5000IE of heparin, the lesion was crossed mainly with an 0.018 V18 Control (Boston) or 0.014 CTO guidewire (Proceed, Abbott) over a support catheter. A filter was not employed in these procedures. The Phoenix Rotational Atherectomy System^TM^ (Philips, San Diego, CA, USA) with a caliber of 1,5 and 1,8 mm was used for the BTK-RA cases. The Phoenix Rotational Atherectomy System^TM^ consists of a catheter with a cutter device at the distal tip. This cutter head rotates at a high speed between 10,000 and 12,000 rounds per minute. This rotational movement of the cutter creates a sucking effect which removes the fragmented atheromatic debris. Due to this effect, the use of a filter is not necessary. The whole system is battery operated and requires a 0.014-inch guidewire to be delivered. The Phoenix Atherectomy 1.5 mm catheter is indicated for BTK-arteries with diameter between 2 and 2.5 mm and requires a 5F introducer sheath. The Phoenix Atherectomy 1.8 mm catheter is indicated for BTK-arteries with diameter between 2.5 and 3 mm and requires a 5F introducer sheath.

Routine use of POBA was additionally performed after BTK-RA. A repeated prolonged angioplasty (3min) was performed in case of recoil or flow-limited dissection. Per protocol, stent implantation with 1:1 oversizing was placed only in cases of persistent flow-limiting dissection or technical failure with high-grade residual stenosis causing >30% lumen narrowing. A balloon-expandable everolimus-coated BTK stent (Xcience Prime, Abbott) was used in ostial short lesions. Postoperatively, all patients were placed on acetylsalicylic acid (ASA) and statin after discharge [14]. If an adjunctive stent was placed, dual antiplatelet therapy was administrated with addition of clopidogrel 75mg daily for 4 weeks. Afterwards, patients were placed in lifelong monotherapy with ASA [14]. 

### 2.5. Follow-Up

All patients were enrolled in a comprehensive 2-year follow-up protocol, emphasizing regular visits at intervals of 3, 6, 12, and 24 months. These visits were meticulously conducted at our dedicated outpatient care facility. During each visit, a thorough clinical examination was administered, encompassing pulse status assessment, ankle-brachial index (ABI) measurements and duplex ultrasonography (DUS), performed by a board certified and experienced sonographer. These systematic examinations provided a framework for the collection of relevant postoperative data in order to conduct a rigorous evaluation of the patients’ vascular health. The combination of pulse status, improvement in ABI, and an adequate sonographic finding with triphasic flow in the targeted vessel was used to assess primary patency.

### 2.6. Statistical Analysis

The statistical analysis was carried out using SPSS 29 (Statistical Package for the Social Sciences, Inc., Chicago, IL, USA). To assess the normality of continuous data, the Shapiro–Wilk test was performed. Continuous variables exhibiting a normal distribution are reported as mean ± standard deviation and were subjected to comparison using the paired Student t-test. Conversely, for continuous variables demonstrating a skewed distribution, the results are presented as median with interquartile range (IQR) and were analyzed using the non-parametric Mann–Whitney U test. Categorical variables underwent examination through Pearson’s chi-squared test to discern any associations or differences among groups. The dynamic aspect of cumulative primary patency over time was captured through Kaplan–Meier curves and survival analysis, providing a visual representation of the data. All statistical tests conducted were two-tailed, aiming for a comprehensive assessment of both sides of the distribution. The threshold for determining statistical significance was set at *p* < 0.05, ensuring a robust and rigorous evaluation of the results.

## 3. Results

Between January 2020 and September 2023, out of 465 patients referred and treated at our vascular department for the treatment of symptomatic PAD, 96 patients with CLTI and isolated BTK-lesions complied with the inclusion criteria for endovascular therapy. Fifty consecutive patients received POBA and were enrolled in group A, while the remaining 46 consecutive patients received BTK-RA and were enrolled in group B. Table 1 summarizes the demographic and preoperative data of all enrolled patients. The entire cohort had a median age of 83, with an interquartile range of 15. Of the enrolled patients, 61% were male. Examining the risk factors associated with PAD, 47% were smokers, 93% had hypertension, 60% experienced hyperlipidemia and 59% had diabetes. Additionally, 51% of patients had a positive history of cardiovascular disease, while 25% suffered from renal disease. The vast majority of treated patients (*n*:88, 92%) presented with minor or major tissue loss due to gangrene, in Rutherford stage 5 or 6. 

Preoperative clopidogrel administration was significantly more frequent in the BTK-RA group (*p* = 0.019), while the POBA group presented significantly lower preoperative ABI (*p* = 0.02), although the absolute difference in ABI between the two groups was only 0.1. Table 2 summarizes the procedural and follow-up data. In our study, it was observed that 52% of cases exhibited occlusive disease. To delve into further detail, within Group A, 48% of cases presented with occlusion of BTK-vessels, while in Group B, this percentage was slightly increased, up to 56%, albeit without reaching statistical significance.

**Figure 1:**
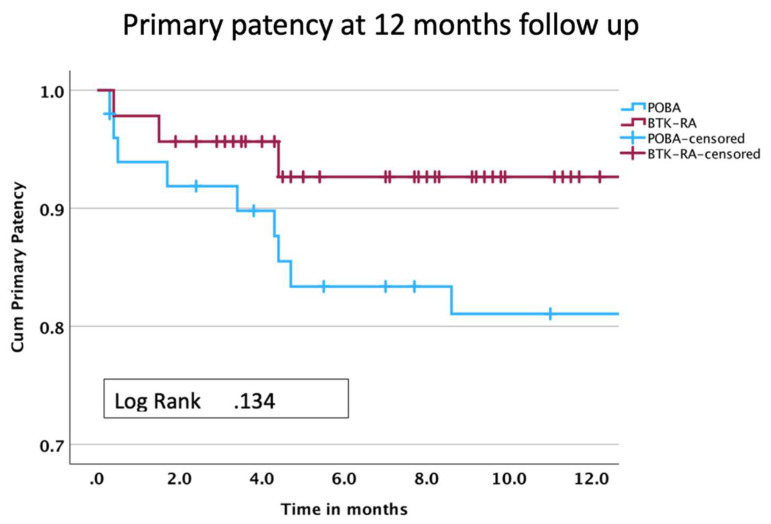
Kaplan–Meier analysis and life table at 12-month follow-up.

The median follow-up for the whole cohort was 8.9 months, with an IQR of 14.7 months. To be more precise, the mean follow-up of POBA was 24.9 months with a standard deviation of 10.5, while the mean follow-up of BTK-RA was 7.9 months with a standard deviation of 4.4. Both techniques had similar technical success (POBA 88% vs. BTK-RA 95.7%, *p* = 0.175), operative time (POBA 64–30 vs. BTK-RA 58–23, *p* = 0.878), intraprocedural complications and bailout stent implantations (POBA 8% vs. BTK-RA 12%, *p* = 0.175). The aforementioned results were independent of the operator’s experience, as both techniques were performed by vascular surgeons certified by the German Society of Vascular Surgery as endovascular specialists as well as by vascular surgeons who were not certified as endovascular specialists (POBA 70% vs. BTK-RA 54%, *p* = 0.114). No instances of intraoperative embolization were recorded. A total of 86% of the cases were performed with antegrade punction (POBA 86% vs. BTK-RA 86%, *p* = 0.891). Only one case, in group B, manifested site access complications. The procedural and follow-up data are summarized in Table 2. The BTK-RA group had significantly higher primary patency rates compared with the POBA group (93.5% vs. 72.0%, respectively, *p* = 0.006), TLR (2.1% vs. 24%, *p* = 0.057), lower in-hospital stay (2.0–3.0 vs. 4.0–6.0 days, respectively, *p* < 0.001) and higher postoperative ABI (0.8–0.2 vs. 0.7–0.1, respectively, *p* = 0.008), though both groups showed a significant increase in ABI postoperatively (*p* < 0.001). Nevertheless, a significantly higher occurrence of postoperative ABI greater than 0.8 was evident in the BTK-RA group compared to the POBA group (67.0% vs. 35.0%, respectively, *p* = 0.005). Due to a significant disparity in the follow-up of the two groups, a survival analysis employing a Kaplan–Meier curve was conducted to thoroughly examine and better illustrate the cumulative primary patency over the follow-up period. The estimated primary patency rates at 12 months (Figure 1, Table 3) showed no significant differences between the two subgroups (Figure 1, *p* = 0.13). Significant differences (POBA *n*: 20, 40%, BTK-RA *n* = 3, 6.5%) were found in minor amputation rates between groups (*p* < 0.001), while the respective limb salvage—meaning salvaging of the ankle—rates were similar in both groups (94.0% vs. 97.8%, *p* = 0.35). 

## 4. Discussion

PAD is a clinical entity which affects more than 200 million worldwide, increasing disability and reducing quality of life [3,15,16]. Since the establishment of endovascular therapy as a first-line treatment, new technologies have been proposed [7]. Atherectomy, with its varieties (rotational, orbital, aspirational), is a promising new therapy which could be used as a vessel preparation technique. 

The outcomes presented here comparing the use of POBA vs. atherectomy-assisted angioplasty in the BTK vessels showed clear clinical benefits with the use of BTK-RA in terms of primary patency, length of hospital stay and postoperative ABI. To our knowledge, this is the first direct and non industry-sponsored comparison between these treatment alternatives in the BTK segment. The results were similar for all operators who performed the procedures, regardless of their experience, showing reproducibility without the need of a prolonged learning curve for at least this type of atherectomy. The intervention time was also similar between the two techniques, also showing less complexity in system preparation and use. This is quite an interesting finding considering that atherectomy is an extra procedural step requiring more time. The reason why the procedural time was the same between the two groups can be attributed to the nature of atherectomy. Atherectomy is not considered to be a standalone therapy but rather represents a vessel preparation technique, which is effective by removing the calcified atheromatic plaques and creating an adequate vessel lumen for the following angioplasty. The current authors suggest that by preparing the vessel before angioplasty, the number of required balloon dilatations is reduced, and, therefore, the procedural time remains the same, regardless the extra procedural step of atherectomy.

Considering the femoropopliteal lesions, drug coated balloon (DCB) angioplasty has been associated with superior primary patency [16]. The literature still contradicts itself as to the efficiency of DCB for the treatment of infrapopliteal lesions. Some meta-analyses show promising patency results while others suggest no significant difference between DCB and POBA [17,18]. Despite the favorable findings in certain studies, there is still no consensus on the recommended use of DCB angioplasty for treating CLTI with infrapopliteal lesions [7]. Since POBA or DCB angioplasty work by pressing the atheromatic material to the arterial wall, it is quite logical to expect that these endovascular techniques would benefit from prior compression of the plaques. In this regard, since becoming commercially available, rotational atherectomy for the treatment of PAD has demonstrated both safety and efficacy [8,11,19] in the femoropopliteal segment [19,20,21,22]. However, considering the BTK vessels, the evidence and published data are still scarce and industry-sponsored. To date, there is only a single systematic review focusing on the general use of atherectomy in the BTK segment showing a potential benefit of the technique, without any specific subgroup analysis, with effectiveness and durability at 12 months of follow-up [23]. However, the available and analyzed articles were without control groups [11,23,24,25]. 

While the BTK-RA group demonstrated notably higher primary patency rates in our study, statistical significance was hindered by the substantial disparity in follow-up durations between the two groups (24.9 months for POBA compared to 7.9 months for BTK-RA). This discrepancy is particularly apparent when examining the Kaplan–Meier curve, where no statistically significant difference in cumulative primary patency emerged during the 12-month follow-up period. Despite the observed trends favoring BTK-RA, the limited follow-up duration cautions against drawing definitive conclusions, emphasizing the need for extended monitoring to establish a more comprehensive assessment of treatment outcomes.

The high technical success rate being independent of the operator’s experience demonstrates the reproducibility of the outcomes, without requiring the extensive experience and prolonged learning curve of other probably complex atherectomy devices. Noteworthily, the interventional time was similar between POBA and BTK-RA, which is extremely beneficial considering that atherectomy is an additional procedural step. The explanation for this is that for the Phoenix device it is not mandatory to use a console as in other rotational atherectomy systems.

The outcomes of our series confirm that the use of atherectomy as vessel preparation debulks the plaque and increases the lumen gain, consequently allowing a better expansion of the following balloon angioplasty and minimizing the risk of early re-stenosis and occlusion. This concept explains the better performance of group B compared to the gold standard of group A. 

Although both techniques resulted in significant improvement of ABI postoperatively, the absolute increase in postoperative ABI was significantly greater (*p* = 0.008) in the BTK-RA group with 0.8 median postoperative ABI compared with 0.7 for the POBA group. Although the statistical difference between postoperative ABIs was significant, the clinical difference between an ABI of 0.8 and an ABI of 0.7 is questionable. The difference between the two values could be attributed to the difference in the preoperative ABI, with the respective values being 0.4 vs. 0.3, which was also statistically significant (*p* = 0.02). A possible explanation for this discrepancy might be the potentially greater implications of the COVID-19 pandemic in the POBA group due to the longer patient recruitment time. It is well established by the VERN COVER study collaborative that the COVID-19 pandemic had a negative effect on vascular surgery worldwide [26]. The suboptimal management of PAD during the pandemic led to an increase of patients with lower ABI measurements and with minor or major tissue loss due to gangrene [26].

Finally, only 6% of the BTK-RA patients required a minor amputation while this rate reached almost 40% for the POBA group. Limb salvage, meaning maintenance of the ankle and heel, was documented for 96% of the total cases without any difference between the two groups. Even if the results in this context are clear and statistically significantly better for the BTK-RA group, the absence of preoperative estimation of possible infection of the foot and its impact in the amputation rate—as no WIFI Classification was documented for the amputated cases—does not allow to draw robust conclusions about the impact of BTK-RA on amputation rates.

### Limitations

The angiographic images presented here were not analyzed by an independent core lab. The absence of a classification to estimate a potential co-existence of infection, especially in the cases in which a minor amputation was performed, blurs the impact of BTK-RA on amputation rates. As per protocol of our clinic, all patients who presented with isolated BTK CTLI between January 2020 and December 2021 received POBA while all patients with isolated BTK CTLI between January 2022 and September 2023 received rotational atherectomy. Therefore, the collection of data regarding lesion morphology and calcification grade was not deemed necessary. This study, conducted at a single center with a relatively modest sample size (96 cases), may yield statistically significant results, however, the limited number of cases may be insufficient to form robust conclusions. Regarding the BTK-RA group, it is important to note that patients are still in the midst of completing their follow-up protocol. As a result, the mid- and long-term outcomes for this group are yet to be fully observed. This ongoing status is also depicted in the Kaplan–Meier curve, where a significant number of patients in the BTK-RA group are marked as censored due to their ongoing follow-up process.

## 5. Conclusions

Rotational atherectomy is a validated technique for the treatment of CLTI with BTK-lesions, demonstrating both safety and effectiveness. Importantly, its application does not demand additional procedural time or specialized experience and training. The use of atherectomy-assisted angioplasty in the BTK segment showed clinical advantages over POBA. In order to establish this promising technology in the arsenal of the modern vascular specialist, multicenter studies with control groups others than POBA and mid- and long-term outcome evaluation are required.

## Figures and Tables

**Table 1:** Demographic and preoperative characteristics.

	Total *n* = 96	Group A *n* = 50	Group B *n*= 46	*p*-Value
Age ^1^	83–15	84–15	82–16.5	0.060
Age > 80 ^2^	54 (56%)	32 (64%)	22 (48%)	0.111
Male ^2^	59 (61%)	29 (58%)	30 (65%)	0.468
Smoking history ^2^	45 (47%)	20 (40%)	25(54%)	0.159
Hypertension ^2^	89 (93%)	44 (88%)	45 (98%)	0.064
Hyperlipidemia ^2^	58 (60%)	26 (52%)	32 (69%)	0.079
Diabetis ^2^	57 (59%)	29 (58%)	28 (61%)	0.775
CAD ^2^	49 (51%)	22 (44%)	27 (59%)	0.147
GFR < 30 mL/min ^2^	24 (25%)	15 (30%)	9 (9%)	0.238
Aspirin ^2^	56 (58%)	27 (54%)	29 (63%)	0.369
Clopidogrel ^2^	8 (8%)	1 (2%)	7 (15%)	0.019
OAC ^2^	7 (7%)	5 (10%)	2 (4%)	0.287
DOAC ^2^	30 (31%)	16 (32%)	14 (30%)	0.869
Stenosis ^2^	46 (48%)	26 (52%)	20 (44%)	0.404
Occlusion ^2^	50 (52%)	24 (48%)	26 (56%)	0.404
Preoperative ABI ^1^	0.4–0.1	0.3–0.2	0.4–0.2	0.002
Rutherford Class 5–6 ^2^	88 (92%)	45 (90%)	43 (93%)	0.538

^1^ Median-IQR. ^2^ *n*, %. Abbreviations: CAD, coronary artery disease; GFR, glomerular filtration rate; OAC, oral anticoagulant; DOAC, direct oral anticoagulant; ABI, anklebranchial index.

**Table 2:** Procedural and follow-up data.

	Total *n* = 96	Group A *n* = 50	Group B *n*= 46	*p*-Value
Endovascular specialist ^1^	60 (62%)	35 (70%)	25 (54%)	0.114
Op duration (minutes) ^2^	59–30	64–30	58–23	0.878
Antegrade punction ^2^	83 (86%)	43 (86%)	40 (86%)	0.891
Technical success ^1^	88 (91.7%)	44 (88%)	44 (95.7%)	0.175
Assisted technical success ^1^	96 (100.0%)	50 (100.0%)	46 (100.0%)	n/a ^3^
Bailout stenting ^1^	8 (8%)	6 (12%)	2 (4%)	0.175
Peripheral embolization ^1^	0 (0.0%)	0 (0.0%)	0 (0.0%)	n/a ^3^
Access site complications ^1^	1 (1%)	0 (0%)	1 (2%)	0.295
Hospital stay (days) ^2^	2.5–5	4–6	2–3	<0.001
Postoperative ABI ^2^	0.8–0.2	0.7–0.1	0.8–0.2	0.008
Postoperative ABI >0.8 ^1^	38 (48%)	16 (35%)	22 (67%)	0.005
30 days MACE ^1^	1 (1%)	1 (2%)	0 (0)	0.335
Mortality ^1^	2 (2%)	2 (4%)	0 (0%)	0.170
Primary patency ^1^	79 (82%)	36 (72%)	43 (93%)	0.006
Reintervention (TLR) ^1,3^	13 (76%) ^3^	12 (85%) ^3^	1 (33%) ^3^	0.052
Minor Amputation ^1^	23 (24%)	20 (40%)	3 (6%)	<0.001
Limb salvage ^1^	92 (96%)	47 (94%)	45 (98%)	0.349

^1^ *n* (%). ^2^ Median-IQR. ^3^ Incidence calculated at patients with failed primary patency, not in the entire cohort. Abbreviations: Op, Operation; ABI, Anklebranchial iindex; MACE, Major adverse cardiovascular event; TLR, Target lesion revascularization.

**Table 3:** Kaplan–Meier analysis and life table at 12-month follow-up.

	0	2	4	6	8	10	12
Total							
Cum Survival (%)		99	98	98	98	97	69
At risk	96	94	93	90	88	87	50
Std error	0.01	0.01	0.01	0.01	0.01	0.02	0.06
Group A							
Cum Survival (%)		90	83	83	81	81	60
At risk	50	44	42	37	36	35	20
Std error	0.04	0.04	0.05	0.05	0.06	0.06	0.09
Group B							
Cum Survival (%)		96	92	92	92	92	92
At risk	46	39	30	22	14	7	3
Std error	0.03	0.03	0.04	0.04	0.04	0.04	0.04

## Data Availability

The data presented in this study are available on request from the corresponding author, Apostolos Pitoulias.
